# Vestibular incisional subperiosteal tunnel access versus coronally advanced flap with connective tissue graft for root coverage of Miller’s class I and II gingival recession: A randomized clinical trial

**DOI:** 10.15171/japid.2019.003

**Published:** 2019-08-31

**Authors:** Saeed Sadat Mansouri, Omid Moghaddas, Narjes Torabi, Katayoun Ghafari

**Affiliations:** ^1^Department of Periodontics, Dental Branch, Islamic Azad University, Tehran, Iran; ^2^Department. of Periodontics, Albert university, Karaj, Iran; ^3^DDS, Private Practice, Tehran, Iran

**Keywords:** Coronally advanced flap, Gingival recession, Root coverage, Subepithelial connective tissue graft, Vestib-ular incisional subperiosteal tunnel access

## Abstract

**Background:**

This study aimed to compare the clinical efficacy of vestibular incisional subperiosteal tunnel access (VISTA) with subepithelial connective tissue graft versus a coronally advanced flap (CAF) with subepithelial connective tissue graft for the treatment of gingival recession defects.

**Materials and Methods.:**

This randomized clinical trial was performed on 24 recession defects that were bilaterally Miller’s class I or II in the maxillary canine and premolar area. One quadrant in each patient was selected randomly to receive VISTA (test group) or CAF (control group) with connective tissue graft. Clinical parameters measured at baseline and at 3- and 6- month postoperative intervals included recession width (RW), recession depth (RD), keratinized tissue width (KTW), clinical attachment level (CAL) and probing depth (PD).

**Results:**

Healing was uneventful in both the test and control groups. At the 6-month follow-up, there was a significant decrease in RD, RW and CAL and an increase in KTW in both the test and control groups. The PD remained unchanged. At 3 and 6 months, no statistically significant differences were found between VISTA and CAF for root coverage and clinical attachment gain. Mean root coverage (MRC) was 70.69% and 67.22% in the test and control group, respectively. VISTA demonstrated higher frequency of complete root coverage (CRC) compared to CAF: 50% vs. 33% (P<0.05). The mean KTW was 2.4±0.7 mm at the test and 2.7±0.8 mm at the control sites (P>0.05)

**Conclusion:**

VISTA, as a minimally invasive approach, can enhance root coverage, KTW and clinical attachment gain; therefore, it can be used as a substitute for CAF with connective tissue graft as a gold standard for root coverage.

## Introduction

Gingival recession is the result of apical migration of soft tissue margin from the cementoenamel junction (CEJ) and presents with breakdown of both the soft and hard tissues.^1^ It can cause functional and esthetic problems such as hypersensitivity, esthetic problems and susceptibility to root caries for patients.^3^ The main reasons for gingival recession include traumatic tooth brushing, improper restoration margins and anatomical conditions such as frenal pull, prominent roots and lack of attached gingiva.^2^ Treatment of gingival recession is a challenge in mucogingival surgery and has a variable rate of predictability. Several surgical techniques, such as free gingival grafts, coronally advanced flaps (CAF), laterally positioned flaps or guided tissue regenerations, have been suggested over the years^4-6^ but according to the available systematic reviews,^7-9^ CAF with connective tissue graft is still considered as a gold standard and yields predictable results in root coverage procedures. Langer and Langer^10^ suggested the use of partial thickness flap with two vertical incisions to cover the connective tissue graft. Nelson described use of a full thickness flap to cover connective tissue graft.^11^ Raetzk^12^ proposed the envelope technique by elevating a partial thickness flap and Zabalegui et al^13^ described a split envelope procedure by using a tunnel approach.

Envelope, tunnel and supraperiosteal techniques might provide more favorable blood supply due to the lack of vertical releasing incisions. In 2011 a new modification of the tunnel approach was introduced by Homa Zadeh,^14^ which is a conservative technique aiming to preserve blood supply and papillary integrity and increase patient compliance. The vestibular incisional subperiosteal tunnel access (VISTA) is a new technique, which is considered to be less invasive, allowing regeneration of gingival tissue by subperiosteal undermining of the soft tissue through a vestibular incision instead of elevating and relocating a flap and it has been reported that it can enhance the revascularization process.^15^

Compared to VISTA, CAF with two vertical releasing incisions might decrease blood supply and jeopardize the predictable complete root coverage outcome. To date, there are no clinical trials available comparing CAF with VISTA for root coverage, and studies carried out on VISTA are scarce. The VISTA requires one single vertical incision in the vestibule underneath mucogingival junction and seems to be an interesting alternative to be evaluated in association with CAF. Thus, the aim of this study was to compare VISTA and CAF, both with connective tissue graft, for root coverage.

## Methods

### Study Design

This study was conducted as a single-center, split-mouth, randomized, clinical trial with the aim of treating Miller’s class I and II gingival recession defects with two different surgical procedures namely VISTA with connective tissue graft (test group) and CAF with connective tissue graft (control group). The study protocol was approved by the Ethics Committee of Islamic Azad University of Tehran. Sample size was calculated at a minimum of eight samples in each group (α<0.01, β<0.05).

### Patient Selection

Patients were selected from the patient pool of the Dental School of Islamic Azad University of Tehran, Iran from May 2015 to March 2016. The patients were included based on the following inclusion criteria: age ≥18 years, presence of Miller’s class I and II defects bilaterally in maxillary canine and premolars with detectable CEJ and full-mouth O'Leary’s plaque index of ≤25% (16). Pregnant or lactating females, smokers, those with systemic disorders contraindicating surgery and diabetes mellitus, those taking medications, those with a history of surgery in the past six months and patients with caries or restorations at the site were excluded. After explaining the procedures and aim of the study to the participants, written informed consent was obtained from them.

Clinical parameters that were evaluated before and after the surgery included complete root coverage (CRC), mean root coverage (MRC), recession depth (RD), recession width (RW), keratinized tissue width (KTW), attachment gain and probing depth (PD), which were compared between the two groups (i.e. VISTA and CAF both with connective tissue grafts).

### Preoperative Clinical Procedures

After periodontal examination, the patients received oral hygiene instructions to eliminate habits related to the etiology of gingival recession. Also, they received dental prophylaxis to decrease their plaque index to <25% (17). They underwent scaling and root planing, and a maintenance program with weekly visits was scheduled for them.

### Intra-examiner Reproducibility

To calibrate the examiner, he measured periodontal parameters in four other patients not included in the study. The patients had one pair of bilateral recession defects around single recessed teeth (RD>2 mm). The examiner repeated the measurements twice with a 24-hour interval. If 90% of the measurements could be reproduced with no more than 1.0 mm difference, calibration was accepted. All the clinical examinations were performed by the same blinded examiner (intra-examiner calibration).

### Data Collection

Two groups of teeth in the same arch, bilaterally, were included in the study. The following clinical parameters were measured with a periodontal probe (UNC15, Hu-Friedy, Chicago, IL, USA) at baseline (before the surgical procedure) and at 3 and 6 months after surgery at the mid-buccal point of the involved teeth: (a) RD was measured in millimeters as the distance from the gingival margin to the CEJ; (b) RW was measured in millimeters at the CEJ; (c) KTW was measured in millimeters as the distance from the mucogingival junction to the gingival margin; (d) PD was measured in millimeters at the distobuccal, mid-buccal and mesiobuccal sites and (e) CAL was calculated in millimeters as RD+PD.^17^

By flipping a coin, it was randomly decided which side would receive the control or test procedure. Each surgical procedure was carried out in a separate session by the same surgeon who was not involved in the clinical measurements.

### Surgical Procedures

In brief, after local anesthesia, the involved root surface was cleaned with a rubber cup and prophylactic paste and root planing was performed. The VISTA was performed with a 1-cm vertical incision made below the mucogingival junction in the vestibule. The subperiosteal flap was undermined with mucoperiosteal elevators and a complete access was provided to undermine the papillae with tunneling instruments (Moghaddas Tunneling Kit; MCT Company, South Korea).

After providing a proper surgical bed in each group of the study, the donor site was prepared on the palate and a single horizontal incision was made.^17^ The incision was made between the distal aspect of the canine tooth and the mid-palatal aspect of the first molar. A connective tissue graft with 1‒1.5-mm thickness was harvested in both groups and pressure was applied on the donor site with wet gauze after graft harvesting and the incision was closed with 4-0 silk sling sutures.

The connective tissue was trimmed and inserted within the subperiosteal tunnel and separately stabilized with sling sutures (5-0 polyglycolic, Vicryl, Supa, Iran). This technique allowed coronal repositioning of the gingival margin, which was then fixed by the coronally anchored suturing technique, and finally the single vertical incision was sutured. Following flap deflection, both groups received scaling and root planing of the exposed areas. Then, the flap was coronally positioned to completely cover the area and fixed with polyglycolic acid sling sutures (5-0 poly glycolic, Vicryl, Supa, Iran).

The CAF procedure was performed by two vertical, divergent releasing incisions made lateral to the recessed area. An intra-crevicular incision was made to meet the releasing incisions and a split-full-split flap was elevated beyond the mucogingival junction. Care was taken to extend the flap to mucogingival junction without perforation (in order not to compromise the blood supply).

Connective tissue graft was trimmed and placed in the recipient site and stabilized with sling sutures. The flap was passively advanced towards the crown to cover both the recession defect and connective tissue. The papillae adjacent to the treated teeth were de-epithelialized and the flap was fixed passively in a coronal position about 1-2 mm above the CEJ. Patients were followed up for six months.

**Figure 1:**
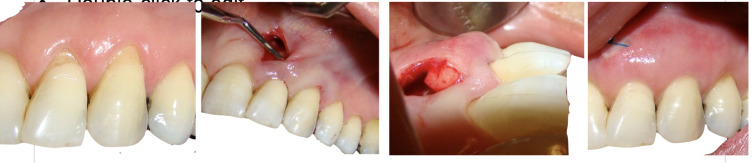
VISTA group; clinical procedure.

**Figure 2:**
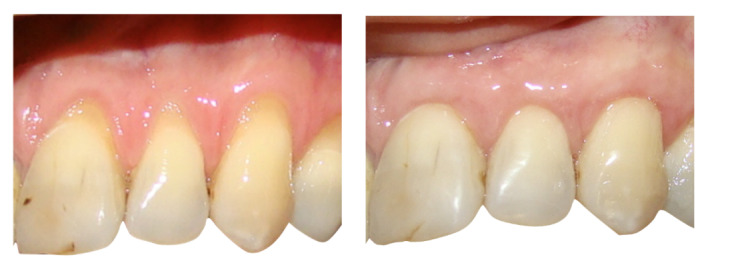
CAF group; baseline and 6-month follow-up.

Figures 1 and 2 present the surgical procedures for the control and test groups, respectively.

### Postoperative Protocol

The patients were instructed to avoid any mechanical trauma or tooth brushing at the surgical site for two weeks. Analgesics (ibuprofen) were prescribed as required and the patients were instructed to rinse their mouth with 0.12% chlorhexidine twice a day for two weeks, and 500 mg amoxicillin TID was prescribed for seven days. Sutures were removed after 14 days. The patients were followed for 3 and 6 months after the surgical procedures. In the first month, the patients were scheduled for professional prophylaxis on a weekly basis. After 30 days, the patients were allowed to gently brush the teeth at the surgical site.

### Statistical Analysis

Statistical analysis was carried out using SPSS 22. Kolmogorov-Smirnovtest was used to evaluate normal distribution of clinical parameters. Paired t-test was used to compare data between the groups at baseline and 3 and 6 months postoperatively. Repeated measures ANOVA was used to compare the differences in clinical parameters in relation to the surgical techniques and time intervals (intra-group difference). Friedman test was applied where repeated measures ANOVA was not applicable. For all the statistical analyses, P<0.05 was considered statistically significant.

## Results

**Figure 3:**
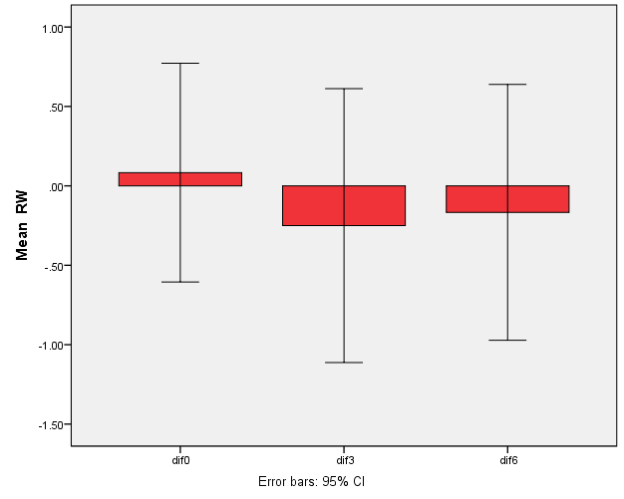
Comparison of the recession width in two techniques at baseline and at three and six months.

**Figure 4:**
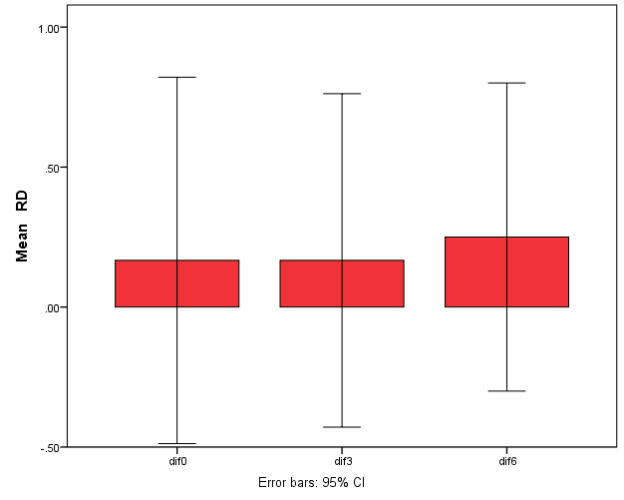
Comparison of the recession depth in the two techniques at baseline and at three and six months.

Twenty-four recession sites were classified as Miller’s class I or II. All the patients completed the follow-up periods. Table 1 presents the clinical data in the test and control groups during the follow-up periods. The RD and RW reductions in both groups were significant compared to baseline (P<0.001) but the differences were not significant between the two groups (Figures 3 and 4).

**Table 1:** Clinical parameters in the test and control groups during the follow-ups

**Clinical parameters**	**Baseline (mm)**	**Three months(mm)**	**Six months(mm)**	**P-value**
**(RD(test**	1.33±2.83	1.02±0.83	1.02±0.83	0.0001
**(RD(control**	1.20±3.00	0.85±1.00	0.79±1.08	0.0001
**P-value**	-0.586	0.551	0.339	
**(RW(test**	1.21±3.25	1.13±1.25	1.13±1.25	0.0001
** (RW(control**	0.88±3.33	1.04±1.00	0.99±1.08	0.0001
**P-value**	-0.795	0.536	0.656	
**(KTW(test**	1.49±2.66	1.50±4.08	1.53±4.00	0.0001
**(KTW(control**	2.63±3.25	2.53±4.66	2.62±4.83	0.0001
**P-value**	-0.253	0.359	0.166	
**(CAL(test**	1.53±4.00	0.90±2.08	0.86±2.25	0.0001
**(CAL(control**	1.34±4.00	0.66±1.91	0.73±2.00	0.0001
**P-value**	-1	0.551	0.389	
**(PD(test**	0.51±1.41	0.45±1.25	0.51±1.41	0.368
**(PD(control**	0.28±1.08	0.28±1.08	0.45±1.25	0.264
**P-value**	--0.110	0.166	0.438	

**Figure 5:**
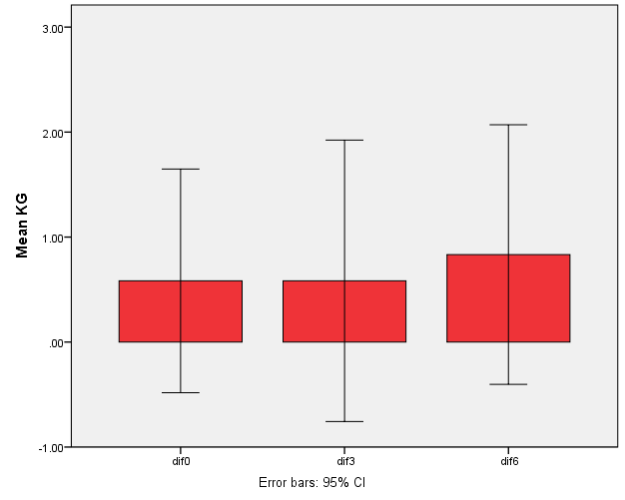
Comparison of keratinized tissue width in the two techniques at baseline and at three and six months.

Keratinized tissue gains in both groups increased significantly compared to baseline (P<0.001) but the difference between the two groups was not significant (P=0.1, Figure 5).

**Figure 6:**
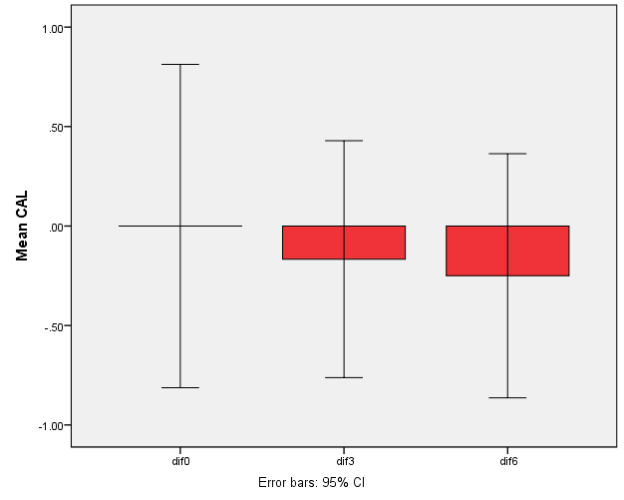
Comparison of clinical attachment levels in the two techniques at baseline and at three and six months.

Clinical attachment level gain in both groups increased significantly compared to baseline (P=0.001) but the difference between the two groups was not significant (P=0.3, Figure 6).

**Figure 7:**
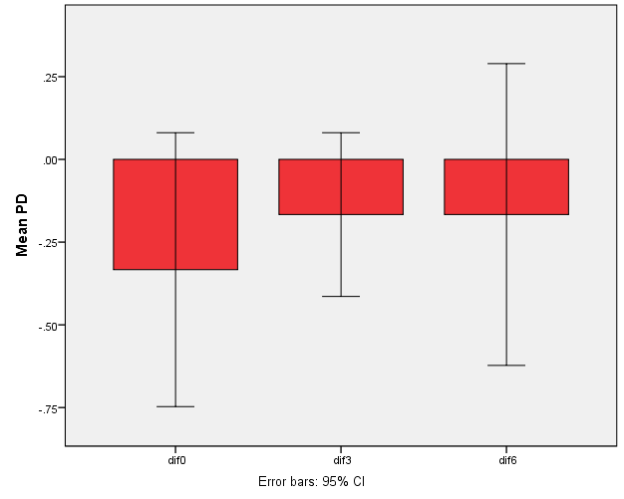
Comparison of probing depths in the two techniques at baseline and at three and six months.

There was no difference between the groups regarding PD reduction compared to baseline (Figure 7).

Table 2 shows the comparison of clinical parameters during the follow-up period.

**Table 2:** Comparison of clinical parameters during the follow-up period

**Clinical Parameters**	**Differences**	**Mean±SD**	**P-value**
**Recession Width**	CAF (0)-VISTA (0)	0.08±1.08	0.595
	CAF (3)-VISTA (3)	-0.25±1.35	
	CAF (6)-VISTA (6)	-0.16±1.26	
**Recession Depth**	CAF (0)-VISTA (0)	0.16±1.02	0.926
	CAF (3)-VISTA (3)	0.16±0.93	
	CAF (6)-VISTA (6)	0.25±0.86	
**Keratinized tissue Width**	CAF (0)-VISTA (0)	0.583±1.67	0.830
	CAF (3)-VISTA (3)	0.583±2.10	
	CAF (6)-VISTA (6)	0.833±1.94	
**Clinical Attachment Level**	CAF (0)-VISTA (0)	0.00±1.20	0.756
	CAF (3)-VISTA (3)	0.16±0.93	
	CAF (6)-VISTA (6)	0.25±0.96	
**Probing Depth**	CAF (0)-VISTA (0)	-0.33±0.38	0.050
	CAF (3)-VISTA (3)	-0.16±0.38	
	CAF (6)-VISTA (6)	-0.16±0.71	

Wound healing was uneventful in all the patients with no graft exposure. Both techniques caused a decrease in gingival RD without a statistically significant difference (P>0.05). The MRC was 70.69% in VISTA and 67.22% in CAF techniques (Table 3). VISTA demonstrated higher frequency of CRC compared to CAF (50% versus 33%).There were no significant differences in gingival thickness between the two groups. The keratinized tissue height increased in the test and control groups by 1.33±0.98 mm and 1.58±1.08 mm, respectively, but the PD remained unchanged. The clinical attachment level gain in both the test and control groups was significant compared to baseline but the differences were not significant between the two groups (Table 4).

**Table 3:** The mean root coverage in the test and control groups (mm/percentage)

**Root coverage technique**	**Root coverage (mm)**	**Root coverage (%)**
**CAF+SCTG**	1.9±0.9	67.22±23.99
**VISTA**	2±1.2	70.69±34/85
**P-value**	0.248	0.383

**Table 4:** Clinical parameters (baseline and 6 months) in the test and control groups (mm)

**Clinicalparameters'technique**	**RW**	**RD**	**KG**	**CAL**	**PD**
**CAF+SCTG**	-2.25±1.13	1.9±0.9	1.58±1.08	-2±1.12	0.16±0.38
**VISTA**	-2±1.20	2±1.2	1.33±0.98	-1.75±1.42	0±0.6
**P-value**	0.555	0.248	0.275	0.571	0.438

## Discussion

Periodontal therapy is performed to eradicate the disease and maintain a functional and healthy dentition and supporting tissues. However, nowadays, periodontal treatments are increasingly directed towards esthetic aspects similar to dental restorations. Gingival recession is defined as apical movement of the gingival margin, leading to root surface exposure, which often causes esthetic problems, increases the susceptibility to root caries and causes dentin hypersensitivity.^2,3,18^ Such defects need to be treated because exposure of root surfaces in the esthetic zone is unpleasant for patients. Complete root coverage up to the CEJ is demanded by many patients complaining about the unaesthetic appearance of their teeth. Several surgical techniques are available for treatment of gingival recessions but according to the available systematic reviews,^9,19,20^ CAF with subepithelial connective tissue graft is the most predictable approach and is considered as the gold standard of root coverage procedures. Also, some modifications of this procedure are available in the literature such as modified coronally advanced tunnel (MCAT)^21^ and modified coronally advanced flap^22^ in conjunction with connective tissue grafts, which have shown predictable results.^23^ VISTA is a modified technique introduced by Zadeh,^14^ and is a minimally invasive approach for root coverage without elevating the papillae and gingival margins and without requiring partial incisions; thus, it maximizes esthetics and enhances tissue healing by not relying on vertical incisions. Our study compared VISTA with CAF and demonstrated similar clinical parameters during six months of follow-up and it was shown that both techniques were predictable in achieving root coverage.

The patients were followed for six months to evaluate the pure effect of surgical procedure without the possible effect of creeping attachment, which usually occurs 6‒12 months postoperatively (24). Creeping attachment was first introduced by Harris (25) and it was reported to consist of about 0.8 mm of tissue migration in the coronal direction but it does not happen all the time.

Several researchers such as Tozum et al^26^ and Zuhr et al^17^ reported favorable results for root coverage in 75.5% and 71.8% of their cases, respectively, when CAF technique was applied. Our study showed 67.22% root coverage at six months, which was consistent with the findings of previous studies using CAF. One concern in CAF is the presence of vertical incisions, which can interrupt blood supply to the site. In addition, possible non-passive flap positioning may also lead to marginal recession, loss of tissue integrity and shrinkage of flap. In our study, the test group (VISTA) showed similar changes in clinical parameters to the control group (CAF) and both procedures showed the same efficacy although VISTA can be considered less invasive. These results are also similar to those reported by Zucchelli et al^27^ comparing CAF and modified CAF, which is an envelope flap with no vertical incisions.

### Complete Root Coverage

Complete root coverage is the ultimate goal of root coverage procedures (17), which can be influenced by multiple factors such as anatomical features, flap design, flap thickness and operator’s experience and skill. In our study, CRC was 50% and 33% in VISTA and CAF groups, respectively. Zucchelli et al,^27^ too, reported proper results regarding CRC in modified CAF compared to CAF (89.3% versus 77.7%). These results can be attributed to the probable benefit of eliminating the need for vertical releasing incisions and better vascularization in such procedures. George and Nisand^28^ showed 50% CRC with the tunnel technique in conjunction with connective tissue graft, which is similar to the findings of our study in the VISTA group. Aroca et al^21^ reported 42% CRC in the group utilizing MCAT plus collagen matrix and 85% CRC when they used MCAT plus connective tissue graft at 12 months. These differences might be due to the use of connective tissue graft instead of collagen matrix in the control group. Zuhr et al^17^ compared the tunnel technique with CAF and reported CRC of 78.6% and 21.6%, respectively. This finding also supports the possible superiority of the results with the use of minimally invasive procedures.^17^

### Mean Root Coverage

In our study, the MRC achieved was 70.69% and 67.22% in the VISTA and CAF groups, respectively; these values seem to be comparable to those reported by Zuhr et al,^17^ who reported 98.4% MRC in the tunnel approach and 71.8% in CAF. The difference between the groups in the study by Zuhr et al can be attributed to the use of connective tissue in the tunnel group, which was not used in the control group. Similar differences were also reported in a study by Aroca et al,^21^ when they compared MCAT with connective tissue (90±18%) and MCAT with collagen matrix (71±21%) and the results were superior in connective tissue group.

A critical technical difference between the VISTA and other tunneling approaches and more classic procedures of gingival augmentation is the amount of coronal advancement of the gingival margin during the procedure. The VISTA approach used in our study combined with a connective tissue graft offers a number of unique advantages for successful treatment of gingival recession defects. The VISTA approach overcomes some of the disadvantages of intra-sulcular tunneling approach and the remote incision reduces the risk of trauma to the gingiva of teeth being treated.

Table 5 demonstrates the comparison of mean root coverage reports between our study and similar studies.

**Table 5:** Comparison of the mean root coverage reports between our study and similar studies

**Study**	**Test**	**Control**	**MRC**	**CRC**
**Current study**	VISTA+CTG	CAF+CTG	70.69% (test) 67.22% (control)	50% (test) 33% (control)
**Aroca et al**	MCAT+CTG	MCAT+CM	71% (test) 90% (control	42% (test) 85% (control)
**Zucchelli et al**	MCAF	CAF	97% (test) 92% (control)	89.3% (test) 77.7% (control)
**Zuhr et al**	TUN	CAF	98% (test) 71% (control)	28.6% (test) 21.4% (control)

### Keratinized Tissue Dimensions

In our study, both groups showed increased KTW (1.33 mm in the VISTA group and 1.58 mm in the CAF group) and the difference in this regard was not significant between the two groups. Similar to the current study, differences were not significant either in the study by Aroca et al^21^ (2.4 mm in MCAT^+^ collagen matrix and 2.7 mm in MCAT^+^ connective tissue). In the study by Zuhr et al,^17^ improvement in KTW was significantly greater in the tunnel group compared to CAF and this difference can be related to the use of connective tissue in the tunnel group and not using it in the control group.^17^

### Clinical Attachment Gain

Clinical attachment gains were 1.75 mm and 2 mm in our test and control groups, respectively; these values were comparable to those in studies by Aroca et al,^21^ (1.9 mm versus 1.4 mm) and Tozum et al^26^ (77.1% versus 56.4%). The possible reason for superior results obtained in our control group compared to other studies might be the use of connective tissue graft in conjunction with CAF.

By careful assessment of the results of studies using the tunnel approach in comparison with ours, it seems that higher mean coverage has been reported in the tunnel approach, which might be related to the elevation of a partial thickness flap in the tunnel approach versus VISTA, which may preserve more of the major blood vessels of the flap in contact with the graft. A higher level of blood supply to the area results in better nourishment of the graft. However, other factors may also play a role in this respect.

Also, differences between our study results and previous literature can be explained by Miller’s classification. In Miller’s classification, the height of the available papilla coronal to the CEJ at the mesial and distal aspects of tooth is not categorized. Only recession defects with sufficient amount of papillary height for flap adaptation were included in our study. Although this point has not been mentioned in most previous studies, its effect on the outcome is unpredictable.

Compared to the VISTA approach, one drawback of the tunnel technique is its limited ability to coronalize the flap, and insufficient graft coverage renders this technique almost unsuitable for recessions with a depth of >5 mm.^21^

On the other hand, in the VISTA technique, all the detachments are subperiosteal and incisions are far from the gingival margin, which minimizes the risk of marginal tissue loss. Subperiosteal tissue detachment also enhances coronalization of the flap and prevents gingival margin stretching when the graft is located beneath the flap.^29,30^ According to the results of our study, there was no significant apical migration of the gingival margin during the follow-up period.

In the original VISTA approach introduced by Homa Zadeh,^14^ absorbable collagen membrane soaked in rhPDGF-BB/TCP composite was used, while in our study, we used connective tissue, which has advantages such as better circulation, lower immunologic reactions and higher cost-effectiveness. Also, compared to previous studies,^9,31,32^ it is obvious that whenever connective tissue is utilized, the long-term stability of the results and the amount of tissue gain are more favorable.

Such researches are technique-sensitive and operative-sensitive; thus, precise execution, methodology, diagnosis, treatment planning and case selection play a significant role in achieving optimal results.

VISTA can be considered as an ideal technique for root coverage that can be easily learned and has benefit over other procedures in terms of esthetics, less tissue manipulation and preserving tissue integrity.

## Conclusion

In conclusion, VISTA, as a minimally invasive approach, was able to treat gingival recession defects and reduce their height and width, yielding results similar to those obtained by the use of CAF as the gold standard procedure for root coverage.
